# 手性多孔有机笼用作气相色谱固定相分离手性和非手性化合物

**DOI:** 10.3724/SP.J.1123.2024.01025

**Published:** 2024-09-08

**Authors:** Bin HUANG, Juan CHEN, Bangjin WANG, Junhui ZHANG, Shengming XIE, Liming YUAN

**Affiliations:** 云南师范大学化学化工学院,云南昆明 650500; School of Chemistry and Chemical Engineering, Yunnan Normal University, Kunming 650500, China

**Keywords:** 气相色谱, 固定相, 手性分离, 多孔有机笼, gas chromatography (GC), stationary phase, chiral separation, porous organic cage (POC)

## Abstract

多孔有机笼(POCs)是一种新型的多孔分子材料,具有明确的分子内腔结构、丰富的主客体识别能力和良好的溶解性,因而在分子识别、气体吸附、传感、催化、色谱分离等多个领域受到广泛关注。本文由四(4-醛基苯基)乙烯和(*R*,*R*)-1,2-环己二胺通过席夫碱缩合反应合成了一种手性多孔有机笼(CPOC),采用静态涂敷法将其涂敷于毛细管柱内表面制备了气相色谱柱,测试了该柱对有机混合物、异构体以及手性化合物的分离能力。结果显示,该柱可以实现4种有机混合物(正构烷烃、芳香烃、正构醇和Grob混合物)中所有组分的基线分离;同时有9种二取代苯位置异构体以及包括结构异构和顺反异构在内的16种有机异构体在该柱上也获得了较好的分离。更重要的是,该柱还具有优秀的手性拆分性能,有12种手性化合物在该柱上得到了拆分,其中5种手性化合物(3-羟基丁酸乙酯、缬氨酸衍生物、谷氨酸衍生物、1,2-丁二醇二乙酸酯和1,2-环氧丁烷)获得了基线分离。将该柱与商品*β*-DEX 120柱的拆分效果相比,该柱可以拆分*β*-DEX 120柱不能拆分的3-羟基丁酸乙酯、缬氨酸衍生物、谷氨酸衍生物、1,2-环氧丁烷、环氧氯丙烷和环氧溴丙烷等6种手性化合物,说明其与*β*-DEX 120柱具有手性拆分互补性。研究表明,CPOC制备的毛细管柱对手性和非手性化合物都具有良好的分离能力。

## Abstract (en)

Porous organic cages (POCs) are a new type of molecular material. The well-defined cavities, abundant host-guest recognition ability, and good solubility of POCs render them attractive for use in various fields such as molecular recognition, gas adsorption, molecular containers, sensing, catalysis, chromatographic separation. In this study, a chiral POC (CPOC) was synthesized via the Schiff base condensation of 4,4',4″,4″'-(ethene-1,1,2,2-tetrayl)tetrabenzaldehyde with (*R*,*R*)-1,2-cyclohexanediamine. CPOC was characterized using nuclear magnetic resonance (NMR) spectroscopy, Fourier transform-infrared (FT-IR) spectroscopy, mass spectroscopy (MS), and thermogravimetric analysis (TGA). The FT-IR spectrum of CPOC showed a strong peak at 1638 cm^-1^, which was attributed to imine (-C=N-) absorption, as well as absorption peaks at 2928 and 2856 cm^-1^, which were attributed to the stretching vibrations of -CH_2_- and -CH-, respectively. MS analysis of CPOC revealed peaks at *m/z*=1801.9797, *m/z*=901.9914, and *m/z*=601.6631, corresponding to [M+H]^+^, [M+2H]^2+^, and [M+3H]^3+^, respectively, and indicating a molecular formula of CPOC (C_126_H_120_N_12_). The TGA curve of CPOC indicated high thermal stability up to 360 ℃; thus, the material is suitable for use as a stationary phase for gas chromatography (GC). CPOC was coated on the inner wall of a capillary column using the static coating method to prepare a GC column. Scanning electron microscopy (SEM) was used to characterize the coating condition of the fabricated column. The SEM images showed that the column had a uniform coating with a thickness of approximately 200 nm. Column efficiency was determined to be 3500 plates/m using *n*-dodecane as a target at 120 ℃. The polarity of the CPOC stationary phase was evaluated using McReynolds constants, which were measured using benzene, 1-nitropropane, 2-pentanone, pyridine, and 1-butanol as probe molecules at 120 ℃. The average McReynolds constant was 152, indicating that CPOC is a moderately polar stationary phase. The ability of the column to separate organic mixtures, isomers, and chiral compounds was subsequently investigated. All components of the four organic mixtures (*n*-alkanes, aromatics, *n*-alcohols, and Grob mixtures) tested achieved baseline separation on the column. In addition, nine positional isomers of disubstituted benzenes were well separated, and seven (*o*,*m*,*p*-nitrotoluene, *o*,*m*,*p*-nitrochlorobenzene, *o*,*m*,*p*-nitrobromobenzene, *o*,*m*,*p*-bromotoluene, *o*,*m*,*p*-dichlorobenzene, *o*,*m*,*p*-chloroaniline, and *o*,*m*,*p*-bromoaniline) achieved baseline separation. Some polar and apolar structural isomers, such as pentanol, dimethylphenol, dimethylaniline, butanol, and C9 aromatic hydrocarbon isomers, were also well separated on the column. Five *cis/trans*-isomers (nerol/geraniol, *cis/trans*-1,3-dichloropropene, *cis/trans*-1,2,3-trichloropropene, *cis/trans*-citral, and *cis/trans*-decahydronaphthalene) were baseline-separated on the column. More importantly, the column successfully separated 12 chiral compounds, indicating good chiral separation ability. Among these chiral compounds, five (ethyl 3-hydroxybutyrate, a valine derivative, a glutamic acid derivative, 1,2-butanediol diacetate, and 1,2-epoxybutane) achieved baseline separation. Six of these chiral compounds (ethyl 3-hydroxybutyrate, the valine derivative, the glutamic acid derivative, 1,2-epoxybutane, epichlorohydrin, and epibromohydrin) could not be separated on a *β*-DEX 120 column but were well separated on the developed column. Moreover, the separation efficiency of 1,2-butanediol diacetate and the isoleucine derivative on this column was better than that on the *β*-DEX 120 column. Separation of the glutamic acid derivative and *o*,*m*,*p*-nitrotoluene was performed before and after the column was used for repeated injections to explore its repeatability. The retention times and selectivity observed after 80, 160, and 500 injections were nearly unchanged compared with those obtained following the first use of the column, indicating that the column has good repeatability. The column was conditioned at 280 ℃ for a certain period to examine its thermal stability. Separation of 3-hydroxybutyrate and *o*,*m*,*p*-nitrochlorobenzene after the column was conditioned at 280 ℃ for 2, 4, or 8 h revealed no obvious changes compared with the first use of the column, indicating that the column had good thermal stability. Thus, CPOC is a stationary phase with good application potential for GC.

大多数情况下,手性药物的对映体中只有一种能够发挥预期的治疗效果,另一种可能是无药效的、有害的或者具有难以预测的副作用^[1]^。市场所销售的很多药物都具有手性并以外消旋体销售,其中一些手性药物的对映体在生物体内表现出的药理活性存在着巨大差异。因此,科研工作者们对于手性拆分领域的研究越来越关注,开发出了酶拆分、膜拆分、色谱拆分等多种手性拆分方法^[2⇓⇓⇓⇓-7]^。色谱拆分法因拆分效率高和适用性广而逐渐成为手性拆分的主要研究方向之一,其中色谱手性固定相(CSP)的开发是该研究的核心内容。气相色谱(GC)是色谱手性拆分的一个重要分支,近些年来许多手性多孔材料被开发为气相色谱CSP,如金属-有机骨架化合物(MOFs)和共价有机骨架化合物(COFs)^[8⇓⇓⇓-12]^。但它们是由强的化学键(共价键或配位键)连接形成无限延伸的多孔框架材料,溶解性差,作为气相色谱固定相时一般只能采用动态涂敷法制备毛细管柱,导致制备的色谱柱重现性、柱效和拆分效果欠佳。

多孔有机笼(POCs)是由分立的笼状分子通过较弱的分子间相互作用自组装形成的一类新型多孔分子材料^[13,14]^,具有稳固的分子内部空腔结构和较好的稳定性,在分子识别、气体吸附、传感、催化、色谱分离等多个领域引起了科研人员的极大研究兴趣^[15⇓-17]^。POCs具有多孔材料的通性,如高的比表面积、有序的孔结构等。与MOFs和COFs等多孔框架材料相比,POCs的典型特点是具有良好的溶解性,可溶解于二氯甲烷、氯仿、甲醇等有机溶剂中,因而更适合用于在均相溶液中进行结构修饰、制备多孔复合材料、涂敷成膜等。POCs作为气相色谱固定相,由于其良好的溶解性,能非常方便地用于静态涂敷法制备柱效高和重现性好的毛细管柱。2015年,Yuan等^[18]^将一种具有三维钻石网状孔通道的手性POC CC3-R涂敷于毛细管内壁制备了气相色谱柱,该柱展现出优异的手性拆分性能,有50多种手性化合物在该柱上获得了较好的拆分,很多手性化合物的拆分效果优于商品*β*-DEX 120柱。此后,相继出现了POCs作为CSP用于GC和CEC拆分手性化合物的报道^[19⇓-21]^。最近,Zhang等^[22]^首次将POCs采用巯基-烯点击反应键合于硅胶表面制备了高效液相色谱手性柱,该柱在正相和反相条件下对醇、酮、胺、酯、有机酸等在内的许多手性化合物表现出优秀的拆分效果,一些手性化合物在该柱上的拆分效果优于商品Chiralpak AD-H和Chiralcel OD-H柱。以上研究结果均表明POCs是一种非常有潜力的色谱CSP,研究开发更多不同种类和结构的POCs作为CSP具有重要的意义。

本文我们将四(4-醛基苯基)乙烯和(*R*,*R*)-1,2-环己二胺通过席夫碱缩合反应合成了一种手性POC(CPOC),将其溶解于二氯甲烷作为固定相,采用静态涂敷法涂敷于毛细管柱内壁制备了GC柱。测试了有机混合物(正构烷烃、正构醇、芳香烃和Grob试剂)、有机异构体(位置、顺反和结构异构体)以及手性化合物在该柱上的分离效果。

## 1 实验部分

### 1.1 试剂与仪器

四(4-醛基苯基)乙烯(纯度>99%,吉林中科研伸科技有限公司)、(*R*,*R*)-1,2-环己二胺(纯度>99%,上海Adamas-beta公司)、三氯甲烷(分析纯,成都市科隆化学品有限公司)、三氟乙酸(纯度>99%,上海Adamas-beta公司)、甲醇(分析纯,成都市科隆化学品有限公司)用于CPOC的合成。混合物分离测试过程所用到的正构烷烃、正构醇、芳香烃以及Grob试剂购自上海Adamas-beta公司,纯度均>99%。位置异构体、顺反异构体和结构异构体均购自上海Aladdin公司,纯度均>99%。所测试的手性化合物购自上海Adamas-beta公司、上海Aladdin公司和日本TCI试剂公司,纯度均>98%。

GC-2014C气相色谱仪(日本Shimadzu公司); Bruker DXR 500 MHz核磁共振波谱仪(瑞士Bruker公司); SDT-650型热重分析仪(美国TA仪器); Nova Nano SEM 450扫描电镜(美国FEI公司); Nicolet iS10傅里叶红外光谱仪和LTQ-Orbitrap XL质谱仪(美国Thermo Scientific公司); TriStar Ⅱ 3flex全自动比表面及孔隙度分析仪(美国Micromeritics公司)。

### 1.2 CPOC的合成

参照文献[23]方法合成CPOC,合成示意图见图1,具体步骤如下:称取四(4-醛基苯基)乙烯(150 mg, 0.34 mmol)和(*R*,*R*)-1,2-环己二胺(76.5 mg, 0.67 mmol)于250 mL烧瓶中,加入50 mL三氯甲烷使固体完全溶解,然后缓慢加入1 μL三氟乙酸,在室温和氮气保护条件下搅拌反应4天。将混合溶液在旋转蒸发仪上真空浓缩至2 mL左右,搅拌并缓慢加入50 mL甲醇,析出黄色固体,过滤后真空干燥得到黄色固体产物CPOC(160 mg)。

**图1:**
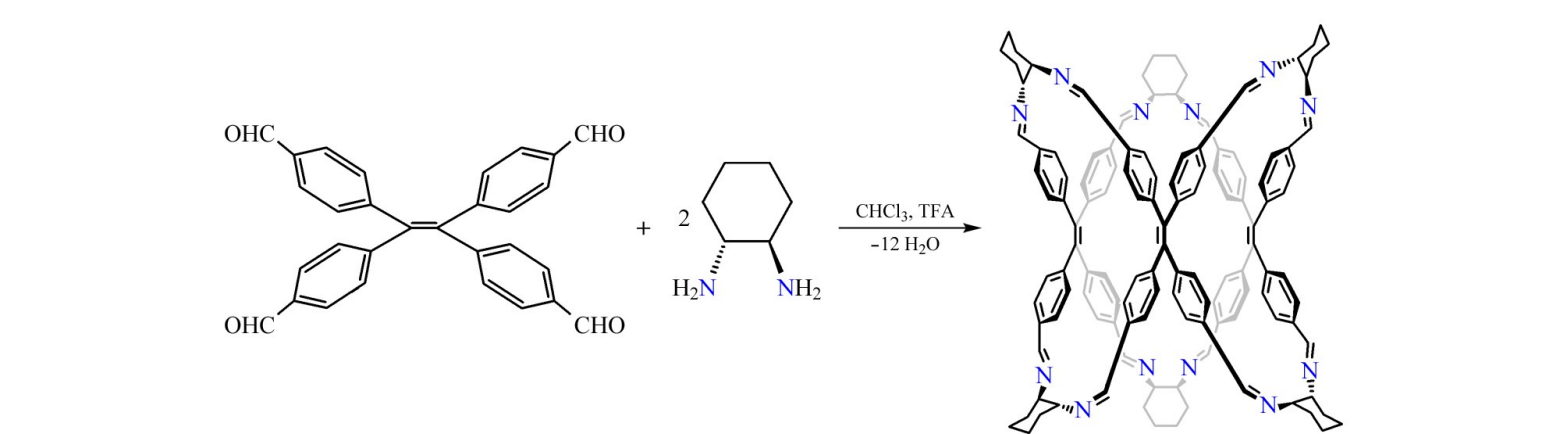
手性多孔有机笼的合成

### 1.3 CPOC毛细管气相色谱柱的制备

首先对空毛细管柱进行预处理:截取一段长15 m的空毛细管柱,依次用1 mol/L NaOH溶液(120 min)、超纯水(60 min)、0.1 mol/L HCl溶液(30 min)和超纯水(120 min)冲洗毛细管柱内壁。最后,将处理好的毛细管柱连接气相色谱仪并在120 ℃下烘干。

采用静态涂敷法制备GC柱:分别配制3 mg/mL的CPOC和聚硅氧烷(OV-1701)二氯甲烷溶液,将两者等体积混溶作为涂敷液,接着将涂敷液泵入上述预处理好的毛细管柱中。当涂敷液从毛细管柱末端流出时用白乳胶密封一端后放置2日。待白乳胶干燥后将毛细管柱放置于39 ℃水浴中,并将未密封的一端连接真空装置以除去柱内的二氯甲烷溶剂,待毛细管内溶剂全部除去即完成柱的涂敷。最后将涂敷完成的毛细管柱连接气相色谱仪进行程序升温处理,具体程序设置如下:起始温度为40 ℃并保持1.5 min,然后以2 ℃/min上升至190 ℃,在190 ℃保持3 h,程序结束后进行后续分离测试。

### 1.4 氨基酸的衍生处理

众所周知,高沸点的分析物无法在GC中直接分析。为了增强分析物的挥发性,获得良好的峰形并提高手性拆分效果,我们将氨基酸通过两步衍生化策略衍生为三氟乙酰基异丙酯衍生物,具体操作步骤如下:将50 mg的氨基酸溶解于5 mL异丙醇-乙酰氯(3∶1, v/v)的混合溶液中,然后将该混合液密封于Teflon反应釜,在110 ℃保持30 min,反应完后用干燥的N_2_吹干除去多余的试剂。将得到的反应产物用5 mL四氢呋喃溶解,然后缓慢逐滴加入1 mL三氟乙酸酐,再次将反应釜密封,在90 ℃保持30 min,反应结束后用干燥的N_2_除去溶剂得到氨基酸的三氟乙酰基异丙酯衍生物。

## 2 结果与讨论

### 2.1 CPOC的表征

#### 2.1.1 核磁共振、红外光谱、质谱和氮气吸附分析

对合成的CPOC进行了核磁共振(NMR)表征,NMR谱图见图2,核磁数据如下:^1^H NMR (500 MHz, CDCl_3_): *δ* 7.84 (s, 6H), 7.78 (s, 6H), 7.36 (d, 12H), 7.02 (d, 12H), 7.00 (d, 12H), 6.94 (d, 12H), 3.31 (s, 6H), 3.15 (s, 6H), 2.18~1.82 (m, 48H); ^13^C NMR (125 MHz, CDCl_3_): *δ* 164.18, 161.73, 144.62, 144.28, 140.15, 135.41, 134.70, 131.45, 131.41, 127.28, 126.78, 73.21, 71.85, 32.45, 32.33, 24.49, 24.31,谱图和数据与文献[23]报道结果一致。对CPOC进行了傅里叶红外光谱分析,如图3a所示,1638 cm^-1^处的强吸收峰是-C=N-的特征峰,2928 cm^-1^和2856 cm^-1^处的吸收峰由-CH_2_-和-CH-的伸缩振动引起。对CPOC进行了质谱分析,如图3b所示,在*m/z*为1801.9797、901.9914和601.6631处的碎片离子峰分别对应[M+H]^+^、[M+2H]^2+^和[M+3H]^3+^峰,这与由分子式(C_126_H_120_N_12_)计算得到的理论数值吻合。我们还对CPOC进行了氮气吸附测试,测试结果如图3c。CPOC的比表面积为515 m^2^/g,孔体积为0.35 cm^3^/g,孔径大小主要在1.2 nm和1.7 nm左右。以上表征证明了CPOC的成功合成。

**图2:**
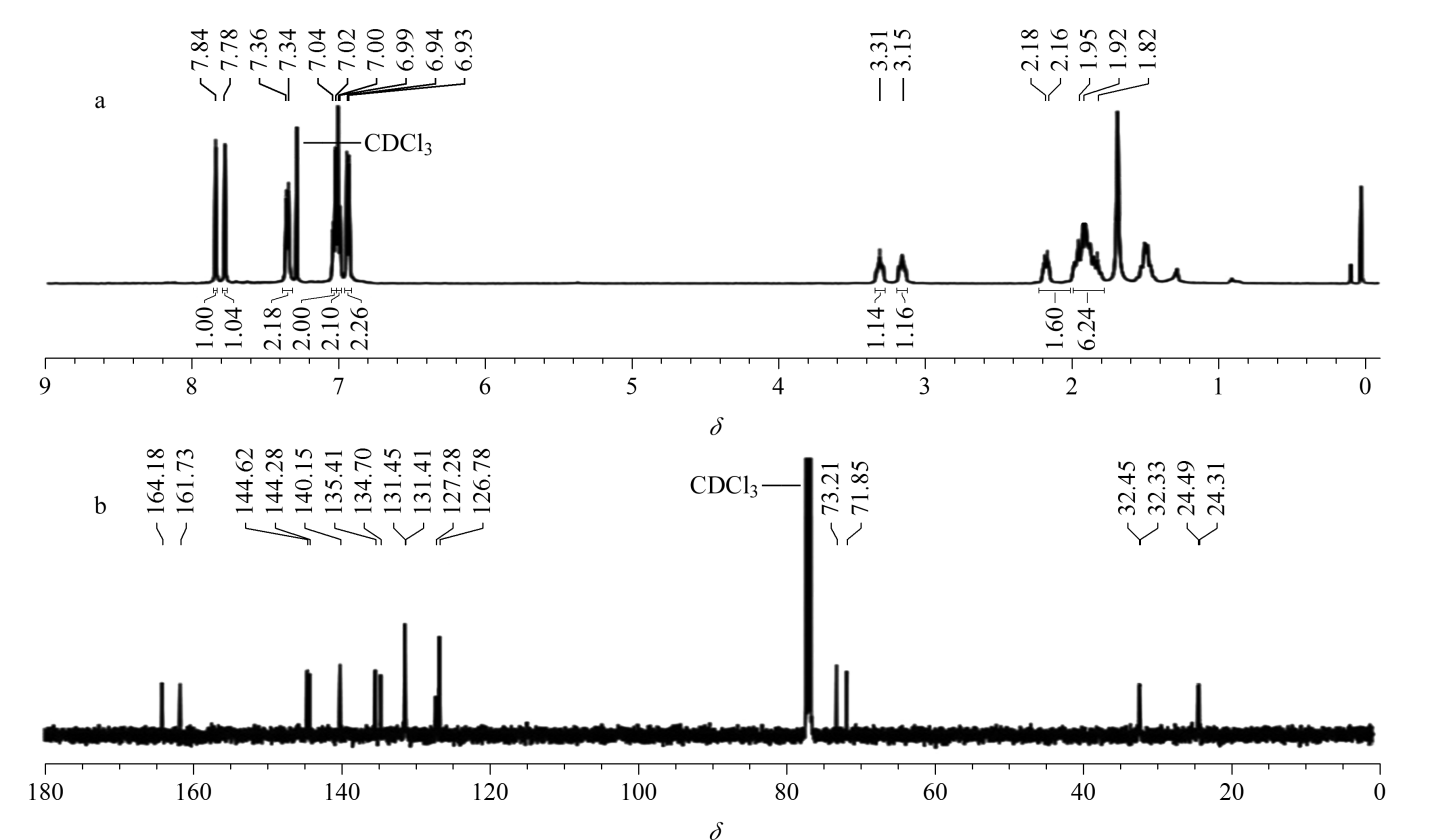
CPOC的核磁谱图

**图3:**
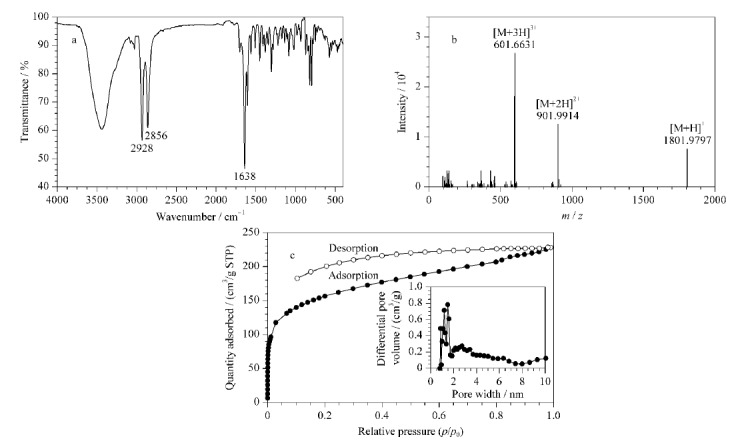
CPOC的(a)傅里叶红外光谱图、(b) ESI-MS质谱图和(c)氮气吸附和孔径分布图

#### 2.1.2 热重分析

固定相是否具有良好的热稳定性在GC分离分析过程中十分关键,我们对CPOC进行了热重分析测试,测试程序:初始温度25 ℃,以10 ℃/min上升至800 ℃。热重分析曲线如图4所示,在360 ℃之前CPOC没有重量损失,始终保持在稳定状态,说明CPOC用作GC固定相具有较好的热稳定性,不易流失。

**图4:**
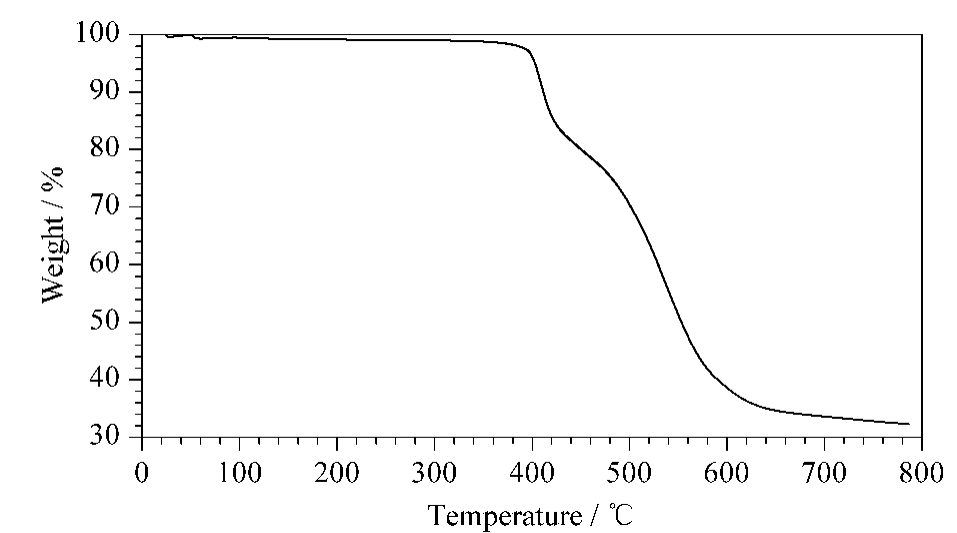
CPOC的热重分析谱图

### 2.2 CPOC毛细管柱的电镜表征、柱效及McReynolds常数

毛细管柱的涂敷厚度和均匀度会直接影响柱效和分离效果。我们将涂敷完成的CPOC毛细管柱和未涂敷的空毛细管柱进行了SEM表征。空毛细管柱的内表面光滑(图5a), CPOC毛细管柱的内表面粗糙,涂层厚度约为200 nm(图5b)。用正十二烷测定了CPOC毛细管柱的理论塔板数,计算得出理论塔板数约为3500块/m。因为CPOC是多孔固体材料,制备的CPOC柱的分离模式是气-固色谱,分析物与多孔固体固定相之间的吸-脱附过程缓慢复杂,容易造成色谱峰展宽。另外,CPOC的涂敷性能和成膜性能也不如传统的气-液色谱固定液(如聚硅氧烷类),因此CPOC的柱效也不如气-液色谱高。选取苯、1-丁醇、2-戊酮、1-硝基丙烷和吡啶等5种分析物测定了该柱的McReynolds常数,得出这5种物质在该柱上的McReynolds常数依次为86、182、116、190、187,平均值为152,表明该柱为中等极性柱。

**图5:**
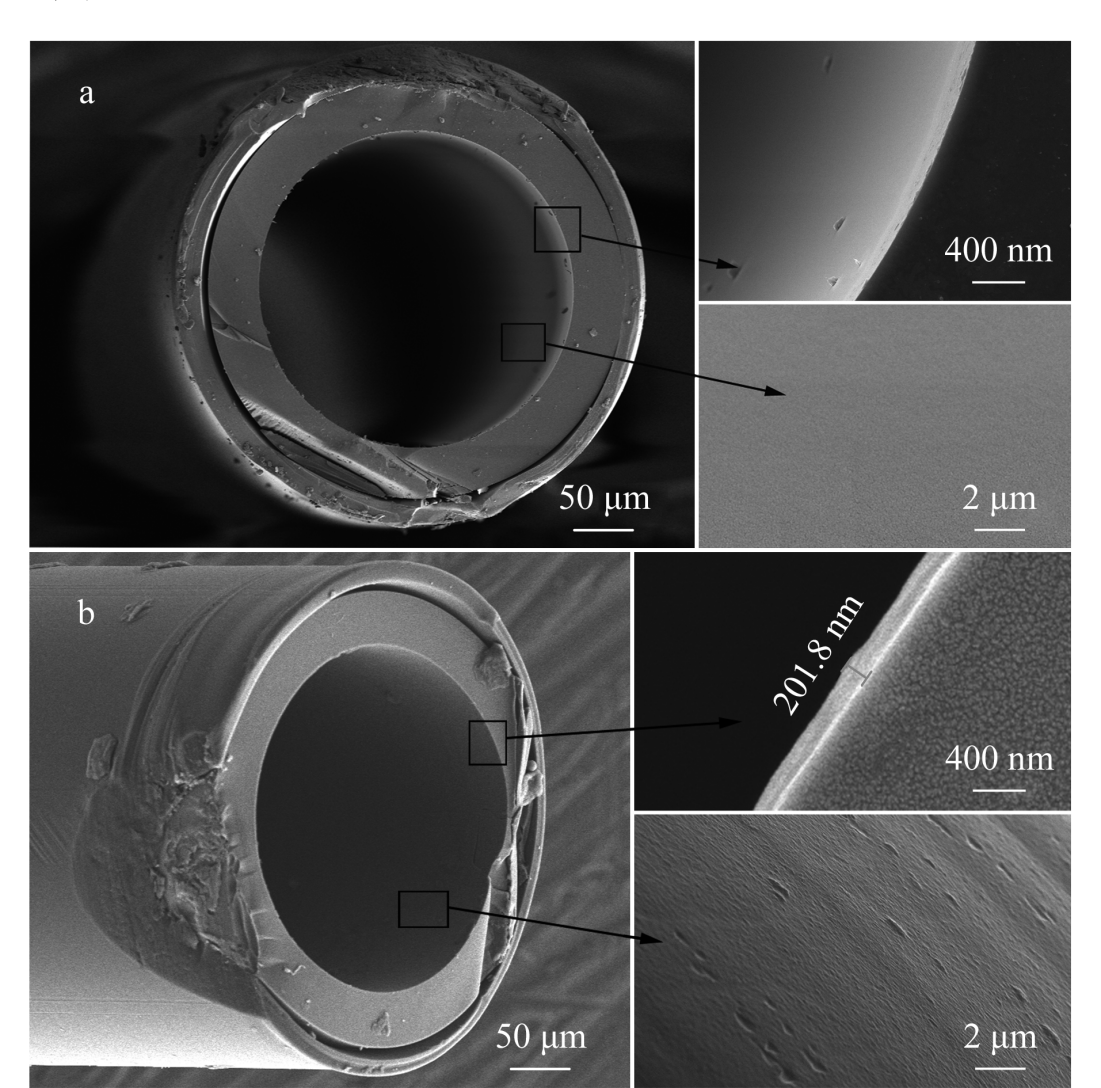
(a)空毛细管柱和(b) CPOC毛细管柱的SEM电镜图

### 2.3 CPOC毛细管柱对混合物的分离

在化工产业中,正构烷烃和芳香烃混合物的分离十分重要。我们测试了9个正构烷烃(正戊烷至正十三烷)混合物以及6个芳香烃混合物(苯、甲苯、乙苯、正丙苯、正丁苯和正戊苯)在CPOC毛细管柱上的分离效果。如图6a和6b所示,这两组混合物中的所有组分都可以在该柱上实现基线分离。醇类物质的分离具有挑战性,通常容易出现色谱峰形拖尾的现象。我们将8个正构醇混合物(*n*-C_1_-OH至*n*-C_8_-OH)在CPOC毛细管柱上进行分离,结果如图6c,所有组分均可被基线分离,色谱峰的峰形尖锐,所有色谱峰的不对称因子都在0.8~1.5范围内,没有出现拖尾现象。Grob试剂可以对色谱柱的性能进行综合评价,为了评估该CPOC毛细管柱的综合性能,我们将2,3-丁二醇、正癸烷、正十一烷、正壬醛、正辛醇、2,6-二甲苯胺、癸酸甲酯、二环己胺和十一酸甲酯组成的Grob混合物在该柱上进行测试(图6d), 9种化合物都可以实现基线分离,色谱峰形良好。以上结果表明该色谱柱具有较好的色谱分离性能。

**图6:**
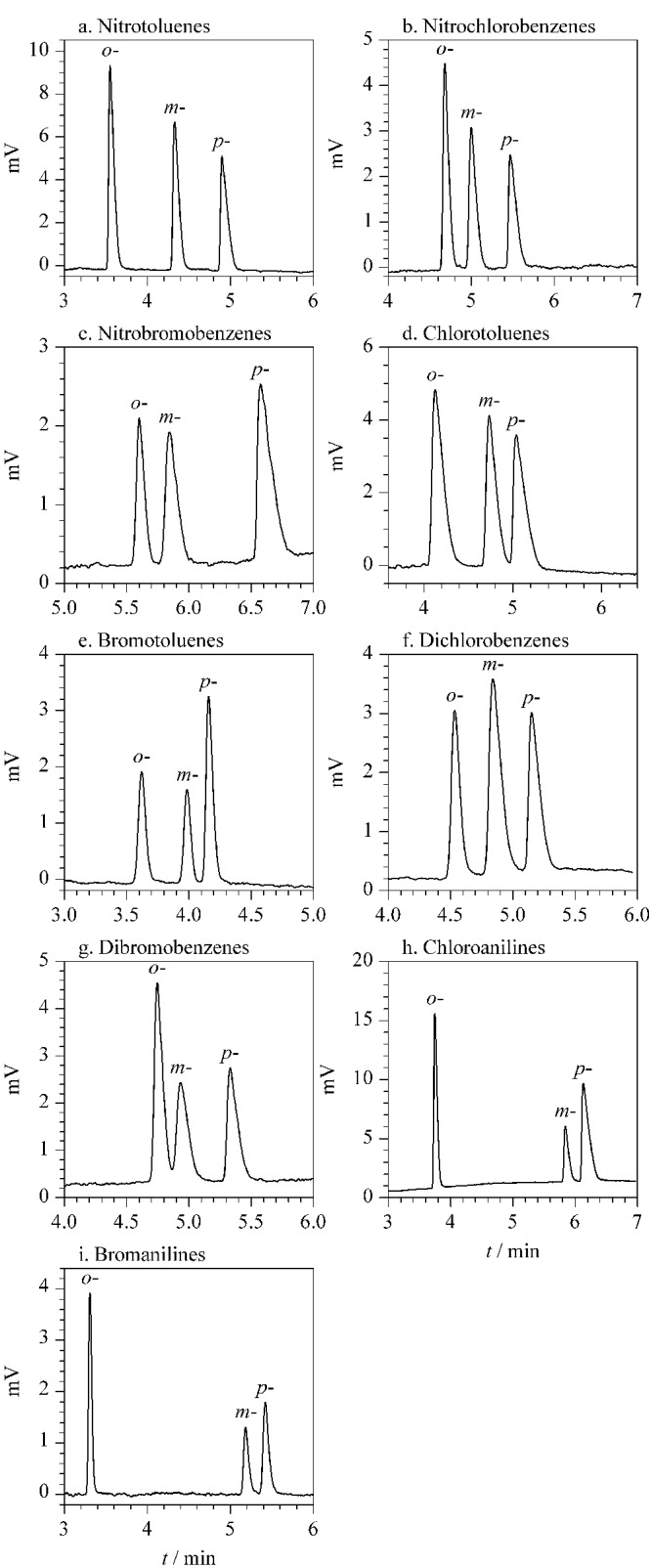
4种有机混合物在CPOC毛细管柱上的分离色谱图

### 2.4 CPOC毛细管柱对有机异构体的分离

有机异构体的化学性质相似,分离较为困难。我们测试了CPOC毛细管柱对多种类型的有机异构体的分离效果,包括二取代苯位置异构体、结构异构体以及顺反异构体。二取代苯位置异构体是重要的工业化学品,如图7和表1所示,共有9种二取代苯位置异构体可以在CPOC毛细管柱上实现分离,其中*o*,*m*,*p*-硝基甲苯、*o*,*m*,*p*-硝基氯苯、*o*,*m*,*p*-硝基溴苯、*o*,*m*,*p*-溴甲苯、*o*,*m*,*p*-二氯苯、*o*,*m*,*p*-氯苯胺和*o*,*m*,*p*-溴苯胺可以实现基线分离。

**图7:**
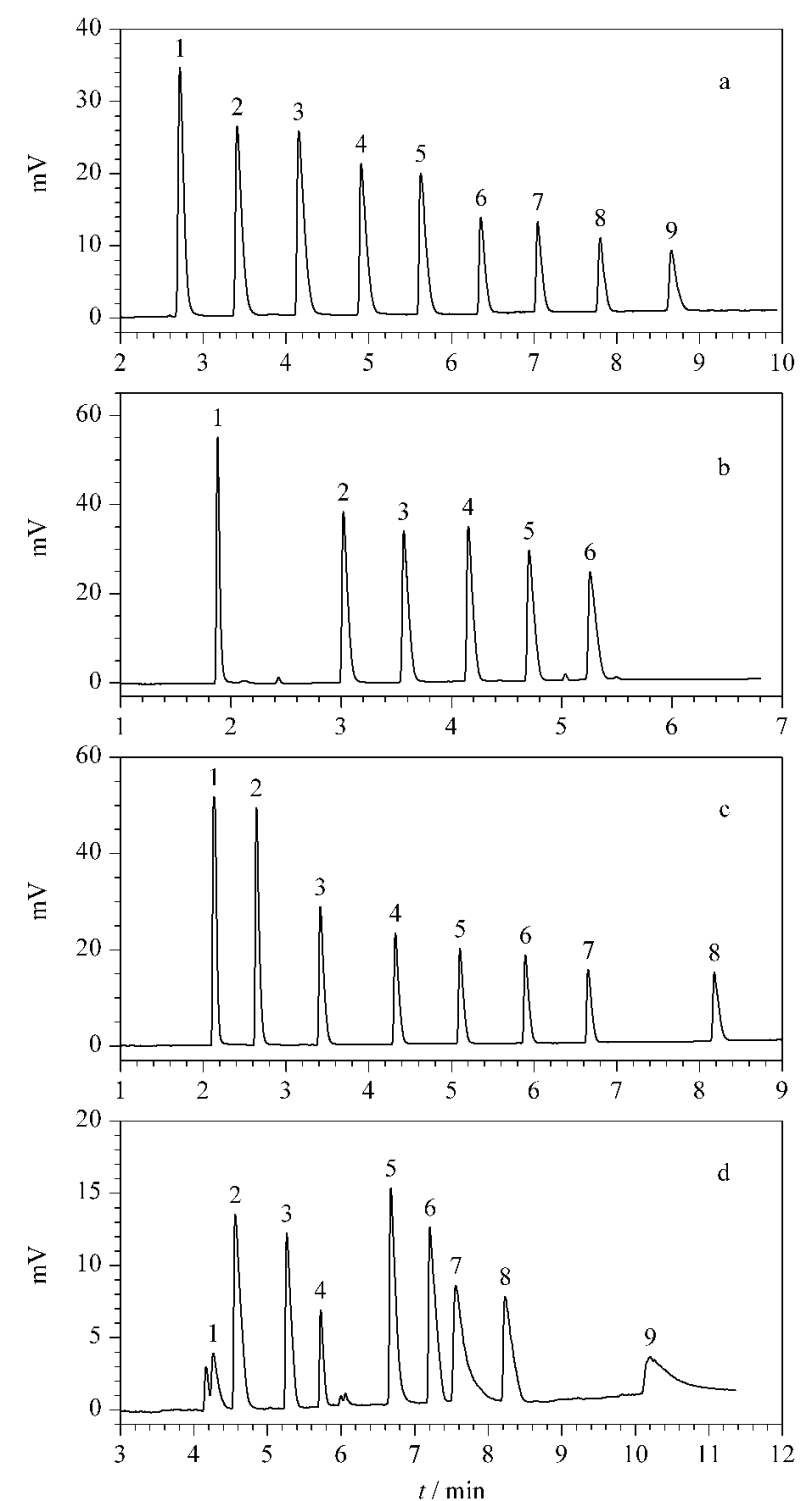
二取代苯位置异构体在CPOC毛细管柱上的分离色谱图

**表1:** 二取代苯位置异构体在CPOC毛细管柱上的分离参数

Positional isomer	*T*^a)^/ ℃	*v*^b)^/ (cm/s)	Separation factor (*α*)			Resolution (*R*_s_)	
			*α*_1_	*α*_2_		*R*_s1_	*R*_s2_
*o*,*m*,*p*-Nitrotoluene	170	15.1	1.38	1.21		6.06	3.80
*o*,*m*,*p*-Nitrochlorobenzene	175	14.8	1.10	1.13		2.22	2.77
*o*,*m*,*p*-Nitrobromobenzene	190	15.9	1.06	1.17		1.57	3.75
*o*,*m*,*p*-Chlorotoluene	130	16.1	1.22	1.10		2.68	1.40
*o*,*m*,*p*-Bromotoluene	155	14.6	1.18	1.06		3.45	1.57
*o*,*m*,*p*-Dichlorobenzene	140	14.9	1.10	1.09		1.93	1.74
*o*,*m*,*p*-Dibromobenzene	170	15.6	1.06	1.12		1.18	2.36
*o*,*m*,*p*-Chloroaniline	180	15.2	1.73	1.06		17.62	1.65
*o*,*m*,*p*-Bromaniline	195	15.1	1.78	1.10		18.39	1.94

a) column temperature; b) linear velocity of carrier gas of N_2_.

强极性和弱极性的结构异构体也可以在CPOC毛细管柱上取得较好的分离效果(图8)。戊醇异构体、二甲苯酚异构体、二甲苯胺异构体和丁醇异构体等4种极性较强的物质中除了2,3-二甲苯酚和3,5-二甲苯酚没有实现基线分离以外,其他3种组分均可以被基线分离(图8a~d)。C9芳烃异构体、C6烷烃异构体以及丁苯异构体等3种弱极性的物质也可以在该柱上实现较好的分离,其中异丙苯/正丙苯、仲丁苯/正丁苯和2-甲基戊烷/正己烷可达到基线分离(图8e~g)。除此之外,还有4种异构体(三氯苯、*α*,*β*-蒎烯、*α*,*β*-紫罗酮和*α*,*β*-萘酚,图8h~k)和5种顺反异构体(橙花醇/香叶醇、*cis/trans*-1,3-二氯丙烯、*cis/trans*-1,2,3-三氯丙烯、*cis*/*trans*-柠檬醛和*cis/trans*-十氢化萘)也可以在该柱上实现基线分离(图8l~p)。

**图8:**
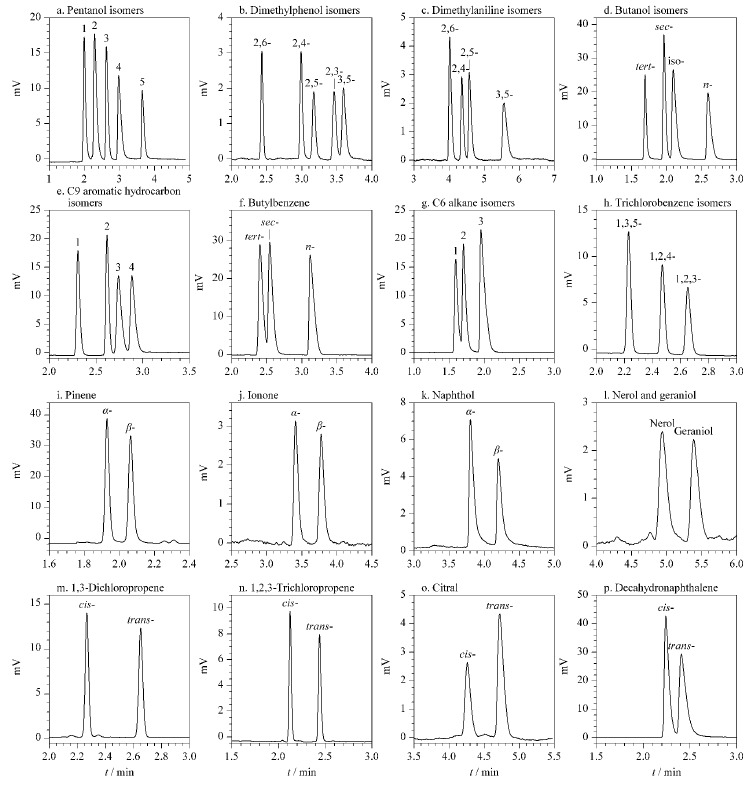
结构和顺反异构体在CPOC毛细管柱上的分离色谱图

有机异构体在常规聚硅氧烷固定相柱(如HP-5、DB-17柱等)上的洗脱顺序通常是按沸点高低的顺序,但在CPOC毛细管柱上的洗脱顺序并不完全遵循沸点顺序,这跟有机异构体的分子几何结构与CPOC分子内空腔匹配度有关,匹配度越好所产生的分子间作用力越强,保留时间则越长。例如,所有二取代苯位置异构体的洗脱顺序都是按*o*-、*m*-、*p*-的顺序,这与CPOC的结构有关。CPOC的分子结构呈管(柱)状,*p*-异构体为线型分子,当其进入CPOC的分子内部空腔时与其柱状结构匹配度高,可产生更强的分子作用力。线型的*p*-异构体更易贴近四(4-醛基苯基)乙烯构筑模块的芳香基面,可与之发生更强的*π-π*作用,因此洗脱时间最长。*o*-异构体的相邻两个取代基对其接近四(4-醛基苯基)乙烯构筑模块芳香基面阻碍作用最大,因此*o*-异构体与CPOC的作用力也最弱,所以也最先被洗脱出来,*m*-异构体洗脱顺序次之。

### 2.5 CPOC毛细管柱对手性化合物的拆分

CPOC毛细管柱对手性化合物也具有较好的拆分效果,图9和表2展示了包括酯类、氨基酸衍生物、环氧化合物等在内的12种手性化合物在该柱上的拆分情况,其中5种手性化合物(3-羟基丁酸乙酯、缬氨酸衍生物、谷氨酸衍生物、1,2-丁二醇二乙酸酯和1,2-环氧丁烷)可以在该柱上实现基线分离。目前,手性毛细管GC柱以环糊精衍生物类为主,*β*-DEX 120柱是一种以全甲基*β*-环糊精为手性固定相的商品化手性柱,具有优秀的手性拆分性能。

**图9:**
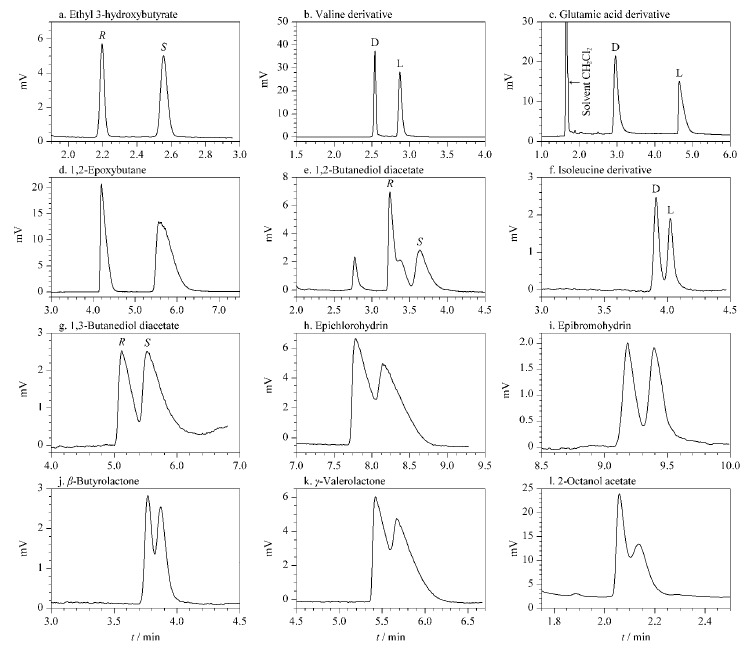
手性化合物在CPOC毛细管柱上的拆分色谱图

**表2:** 手性化合物在CPOC毛细管柱上的拆分数据

Racemate	CPOC-based GC column						*β*-DEX 120 column					
	*T*/ ℃	*k*_1_	*α*	*R*_s_	*v*/(cm/s)		*T*/ ℃		*k*_1_	*α*	*R*_s_	*v*/(cm/s)
Ethyl 3-hydroxybutyrate	160	0.86	1.30	5.46	15.1		100		4.25	1.00	-^b)^	13.5
Valine^a)^	155	0.81	1.22	4.87	15.3		95		5.21	1.00	-^b)^	15.1
Glutamic acid^a)^	185	1.01	1.29	7.16	14.9		160		6.66	1.00	-^b)^	15.6
1,2-Epoxybutane	70	1.79	1.21	5.01	14.6		40		1.54	1.00	-^b)^	14.3
1,2-Butanediol diacetate	175	1.16	1.08	1.77	16.2		135		2.64	1.02	1.11	15.5
Isoleucine^a)^	165	1.61	1.05	1.30	15.3		95		9.80	1.02	1.03	15.0
1,3-Butanediol diacetate	180	2.41	1.04	0.86	14.1		140		2.65	1.03	1.52	15.0
Epichlorohydrin	90	4.19	1.02	0.70	13.9		65		2.82	1.00	-^b)^	14.3
Epibromohydrin	95	5.12	1.06	1.18	15.0		70		5.74	1.00	-^b)^	14.3
*β*-Butyrolactone	110	1.51	1.04	0.74	14.6		80		3.45	1.02	1.15	13.5
*γ*-Valerolactone	120	2.62	1.02	0.64	15.5		105		4.95	1.03	1.45	13.5
2-Octanol acetate	160	0.75	1.01	0.60	15.9		110		3.87	1.10	5.25	14.3

a) trifluoroacetyl isopropyl ester derivative; b) cannot be separated; *k*_1_: retention factor of the first enantiomer.

这12种手性化合物也在*β*-DEX 120柱上进行了拆分测试,所得的拆分数据与CPOC毛细管柱进行了对比(表2)。如表2所示,有6种手性化合物(3-羟基丁酸乙酯、缬氨酸衍生物、谷氨酸衍生物、1,2-环氧丁烷、环氧氯丙烷和环氧溴丙烷)不能在*β*-DEX 120柱上实现拆分,但是可以在CPOCs毛细管柱上得到拆分。除此之外,1,2-丁二醇二乙酸酯和异亮氨酸衍生物在CPOC毛细管柱上的分离度(*R*_s_)大于*β*-DEX 120柱。结果表明,所制备的CPOC是一种具有显著手性识别能力的CSP,并与*β*-DEX 120柱具有手性拆分互补性,能拆分*β*-DEX 120柱不能拆分的一些手性化合物。

CPOC是由四(4-醛基苯基)乙烯和(*R*,*R*)-1,2-环己二胺通过席夫碱缩合反应制备的笼型分子,分子中心具有内腔结构,顶部和底部各具有一个三角形状的孔窗口,孔尺寸为14.352 Å(图10a, 10b)。分析物的分子尺寸均小于CPOC的孔窗口,可通过窗口进入到CPOC的内部空腔,一些典型分析物的分子尺寸大小如图10c所示。分析物进入到CPOC的分子内部空腔,可与之发生丰富的主客体包合、氢键、*π-π*、偶极-偶极等相互作用,从而实现手性分离。例如,对于一些氢键供体的手性分子(如3-羟基丁酸乙酯、氨基酸衍生物等),此时分析物与CPOC的氮原子之间的氢键作用在手性分离过程中起着重要的作用,这些化合得到了较好的分离。除此以外,它们之间的氢键、*π-π*、偶极-偶极等相互作用力也对手性分离起重要作用。

**图10:**
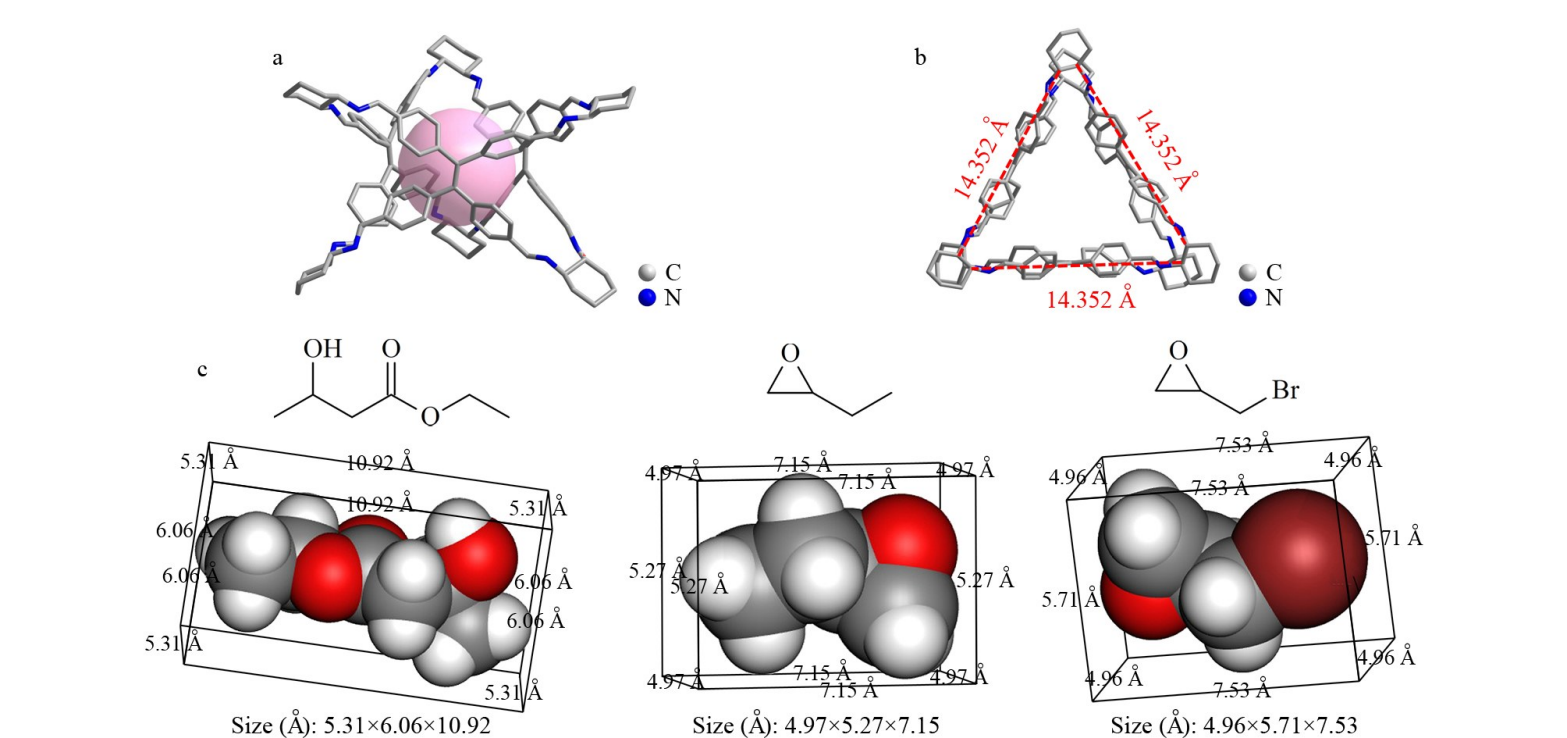
CPOC分子结构和手性化合物分子尺寸图

### 2.6 CPOC毛细管柱的重复性和热稳定性考察

选取谷氨酸衍生物和硝基甲苯进行柱的重复性测试。在色谱柱使用之初对这两种物质进行分离测试,然后在柱分别经过80、160和500次进样使用后,再次对这两种物质进行分离测试,将得到的色谱图进行对比。如图11所示,CPOC毛细管柱在使用初期对两种物质的分离效果与经过多次进样使用后的分离效果几乎没有差别,由此可见该柱具有良好的重复性。CPOC毛细管柱的热稳定性考察选取3-羟基丁酸乙酯和硝基氯苯作为分析物,将该柱在280 ℃高温条件下保持2 h后对分析物进行分离,同理保持4 h和8 h后再次对分析物进行分离,将得到的色谱图进行对比。如图12所示,长时间的高温处理并没有影响CPOC毛细管柱的分离能力,高温处理前后得到的色谱图分离效果基本没有明显变化,表明该柱热稳定性良好。

**图11:**
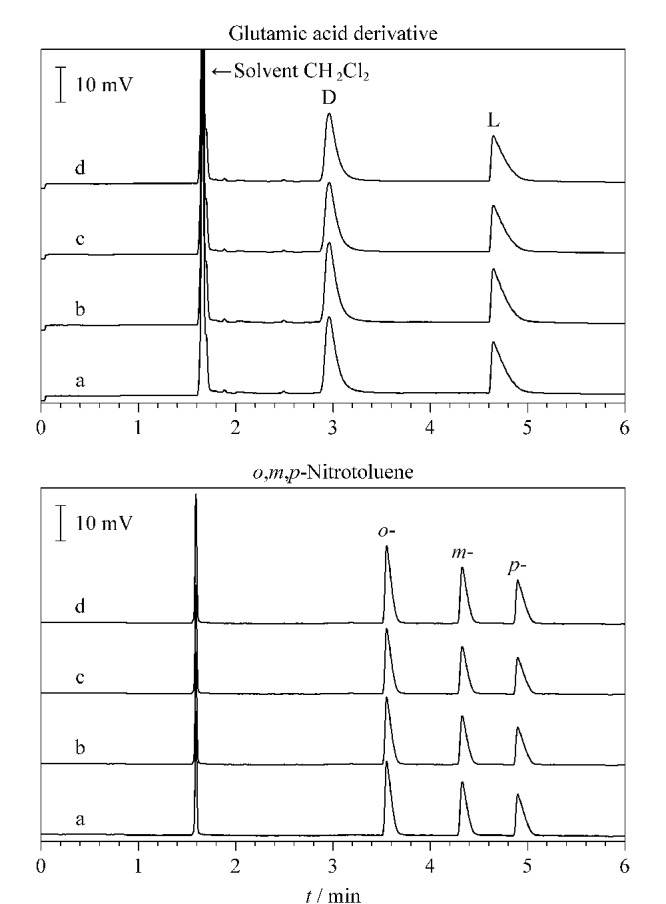
CPOC毛细管柱的重复性

**图12:**
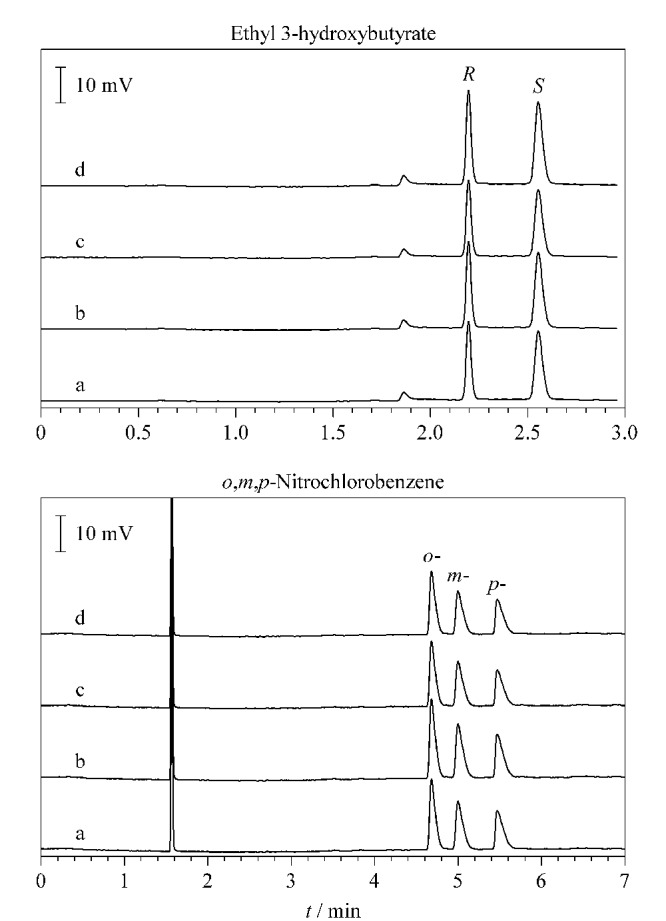
CPOC毛细管柱的热稳定性

## 3 结论

本文合成了一种手性多孔有机笼,将其均匀涂敷于毛细管柱内壁制备了CPOC毛细管柱。该柱可以分离正构烷烃混合物、正构醇混合物、芳香烃混合物、Grob混合物和一些有机异构体(包括位置异构体、顺反异构体和结构异构体)。另外,该柱还具有优秀的手性拆分能力,可拆分包括酯类、氨基酸衍生物、环氧化合物等在内的12种手性化合物。该柱与商品*β*-DEX 120柱具有良好的手性拆分互补性,其中有6种手性化合物能被该柱拆分而不能被*β*-DEX 120柱拆分,还有2种手性化合物的分离度优于*β*-DEX 120柱。CPOC毛细管柱展现出了优秀的分离性能,其在分离分析领域具有潜在的应用价值。
